# Challenges and Opportunities in Disease Forecasting in Outbreak Settings: A Case Study of Measles in Lola Prefecture, Guinea

**DOI:** 10.4269/ajtmh.17-0218

**Published:** 2018-03-12

**Authors:** Matthew Graham, Jonathan E. Suk, Saki Takahashi, C. Jessica Metcalf, A. Paez Jimenez, Vladimir Prikazsky, Matthew J. Ferrari, Justin Lessler

**Affiliations:** 1 Johns Hopkins Bloomberg School of Public Health, Baltimore, Maryland;; 2 Oxford University Clinical Research Unit, Ho Chi Minh City, Vietnam;; 3 European Centre for Disease Prevention and Control, Solna, Sweden;; 4 World Health Organization, Geneva, Switzerland;; 5 Department of Ecology and Evolutionary Biology, Princeton University, Princeton, New Jersey;; 6 Woodrow Wilson School, Princeton University, Princeton, New Jersey;; 7 Department of Biology, Center for Infectious Disease Dynamics, Pennsylvania State University, University Park, Pennsylvania;; 8 Department of Statistics, Pennsylvania State University, University Park, Pennsylvania

## Abstract

We report on and evaluate the process and findings of a real-time modeling exercise in response to an outbreak of measles in Lola prefecture, Guinea, in early 2015 in the wake of the Ebola crisis. Multiple statistical methods for the estimation of the size of the susceptible (i.e., unvaccinated) population were applied to weekly reported measles case data on seven subprefectures throughout Lola. Stochastic compartmental models were used to project future measles incidence in each subprefecture in both an initial and a follow-up iteration of forecasting. Measles susceptibility among 1- to 5-year-olds was estimated to be between 24% and 43% at the beginning of the outbreak. Based on this high baseline susceptibility, initial projections forecasted a large outbreak occurring over approximately 10 weeks and infecting 40 children per 1,000. Subsequent forecasts based on updated data mitigated this initial projection, but still predicted a significant outbreak. A catch-up vaccination campaign took place at the same time as this second forecast and measles cases quickly receded. Of note, case reports used to fit models changed significantly between forecast rounds. Model-based projections of both current population risk and future incidence can help in setting priorities and planning during an outbreak response. A swiftly changing situation on the ground, coupled with data uncertainties and the need to adjust standard analytical approaches to deal with sparse data, presents significant challenges. Appropriate presentation of results as planning scenarios, as well as presentations of uncertainty and two-way communication, is essential to the effective use of modeling studies in outbreak response.

## INTRODUCTION

**Figure 1.**
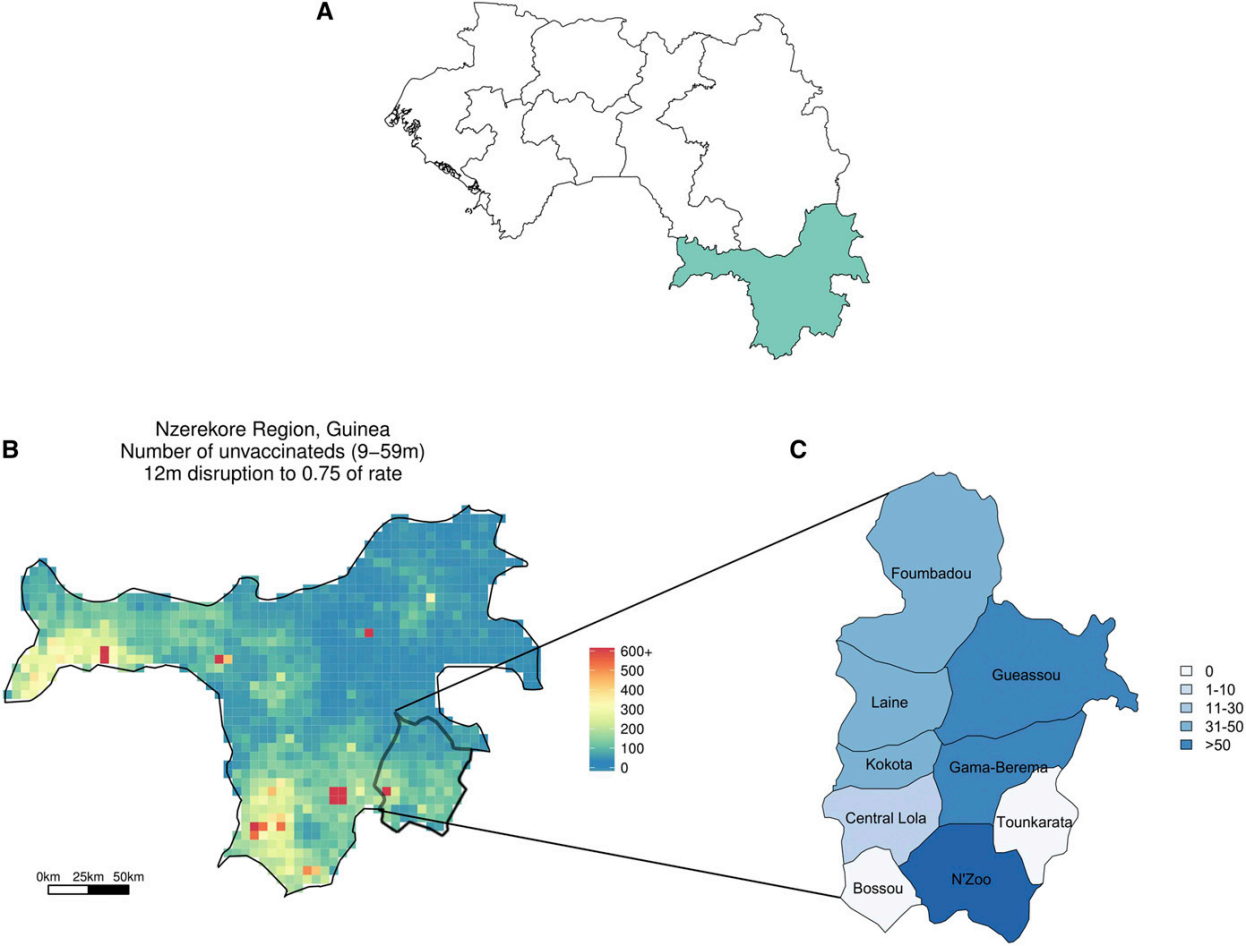
(**A**) Map of Guinea, with regions outlined and the Nzérékoré region highlighted. (**B**) Zoomed in, we see the number of susceptible individuals in the Nzérékoré region of Guinea if a 25% interruption of measles vaccination due to the impact of Ebola on the healthcare system is assumed. Lola prefecture is in the lower right-hand corner of the map and is seen in (**C**) with labeled subprefectures. The color of each subprefecture coincides with the number of reported measles cases up to week 13 of 2015. This figure appears in color at www.ajtmh.org.

Between January 23 and April 4, 2015 (weeks 4–13 of the year), 284 cases of measles were identified in Lola, a prefecture of approximately 180,000 people in southeast Guinea within the Nzérékoré region (Figure 1). Given healthcare system disruptions caused by the Ebola outbreak, there was concern that reductions in measles vaccination may have increased susceptibility in the younger population.^1^ A supplementary immunization activity, aimed at decreasing measles susceptibility, was planned for Guinea in 2014. However, this campaign was interrupted by the Ebola outbreak and never reached Lola prefecture. In addition, within the Nzérékoré region of Guinea, measles vaccination coverage has been relatively low (reaching only 61% of children aged 9–59 months in 2012^2^), suggesting that a large proportion of the population aged less than 5 years was susceptible to a measles outbreak. These factors raised concerns that these 284 reported cases heralded a large and potentially deadly measles outbreak (e.g., the estimated case–fatality ratio of measles cases in Africa was 3.7%).^3^

At the request of staff from the European Center for Disease Prevention and Control (ECDC), who were coordinating the Global Outbreak Alert and Response Network field team based in Lola, an informal collaboration with the Johns Hopkins School of Public Health, Princeton, and Penn State was established to estimate measles susceptibility in Lola and to forecast the course of the outbreak. Data collected by local partners were used to create rapid estimates of population susceptibility to measles and to develop scenario-based forecasts of future incidence aimed at informing the local public health response. Initial analyses and forecasts were based on case data from January 23 to March 29, 2015 (week 13 of the year), which we term the Week 13 report; a second set of forecasts was based on data through April 26, 2015 (week 17), termed the Week 17 report.

This response was particular in its opportunities and challenges. Because of the efforts of the local public health team, it is believed that measles incidence in N’Zoo subprefecture of Lola was nearly completely observed, providing a rare opportunity for the analysis of disease dynamics.^4^ However, data from surrounding areas were sparse and assumed to be underreported, with very little of the epidemic observed at the time the initial predictions were made (Figure 1). Furthermore, the Week 13 report and the Week 17 report contained different numbers of cases for the weeks that were included in both data sets, presenting an additional challenge to generating accurate results. The data from the Week 17 report, up to and including week 13, are termed the corrected Week 13 report.

Here, we report the results of our analyses in support of an ongoing public health response. We evaluate the accuracy of round 1 predictions in forecasting later incidence and attempt to evaluate the extent to which inaccuracies could be attributed to data issues versus model misspecification. We also detail caveats and complexities related to this real-time modeling effort.

## METHODS

The aims of this analysis were 2-fold: to estimate the level of measles susceptibility in the population and to forecast the number of expected future cases in Lola prefecture. Initial analyses focused on N’Zoo subprefecture as, along with a high level of observation of the epidemic, it had the longest history of case reports, likely improving the accuracy of analysis relative to other locations.

### Susceptibility estimation.

Three methods were used to generate estimates of measles susceptibility in children between the ages of 9 months and 5 years. The first was based on the work by Orenstein et al.,^5^ which uses the proportion of cases occurring in vaccinated individuals and vaccine efficacy (VE) to estimate the proportion of the population vaccinated (PPV) and immune, based solely on case data (see Supplemental Information). VE was assumed to be between 86% and 97%, reflecting reasonable bounds on the efficacy of one dose of measles vaccination across several studies.^6–8^

The second method combined data from the Demographic and Health Surveys (DHS)^9^ and demographic models to estimate the PPV, given reductions in vaccination rates stemming from the Ebola epidemic, as developed by Takahashi et al.^1^ Based on data collected by Médecins Sans Frontières throughout the Ebola-infected regions, it was assumed that vaccination rates decreased by 25% over the previous 12 months. Subsequent analysis of the decrease in routine immunization in Liberia showed drops in Ebola-affected areas of between 33% and 58%, suggesting that a decrease of 25% may be an underestimate of the drop in vaccination rate.^10^ This was combined with DHS data from the 2012 survey in Guinea and spatially explicit data on birth rates and population distributions from the WorldPop project^11^ to estimate the level of susceptibility in the population at the beginning of the measles outbreak (see Supplemental Information).

The third method used Markov chain Monte Carlo methods to fit a seasonally forced, time-series susceptible–infected–recovered model (TSIR) to the initially observed course of the epidemic. For N’Zoo subprefecture, specific values of the basic reproductive number *R*_0_, consistent with those previously estimated for measles,^12,13^ were assumed in the initial analysis: *R*_0_ values of 8, 12, and 18 were considered. Susceptibility levels were estimated based on the relationship between these assumed *R*_0_ values and the observed epidemic curves. Four independent chains of length 5,000 were run using RStan^14,15^ and convergence was assessed using Gelman and Rubin’s^16^ Rhat statistic. We assumed that the number of infections in a given week was dependent on the number of infections 2 weeks earlier, that is, the force of infection on the population at a given time depends on the number of infections 2 weeks earlier. This approximates the serial interval of measles (the length of time between successive cases in a transmission chain).^17,18^ Seasonality was modeled by the equation β(*t*) = mean(β)(1 + α cos(2π*t*)), where β is the transmission parameter and α is the amplitude of the seasonal effect on transmission and is related to *R*_0_ via the next-generation matrix of the model.^19^ Initial analysis fixed *R*_0_ and the seasonality effect, α, in the model, whereas retrospective analysis let *R*_0_ and α be fit via the TSIR model. In the other subprefectures, measles was assumed to be underreported; therefore, an observation parameter δ was also fit, translating numbers of infections to numbers of reported cases. Susceptibility projections for N’Zoo were used as prior distributions when fitting other locations. Additional model details are available in the Supplemental Information.

### Epidemic projections.

We projected measles cases into the following weeks via stochastic forward simulation of the epidemic. It was assumed that the force of infection in a given week was based on the mean of the number of infections in the previous 2 weeks. The basic model for forward predictions was the same for all subprefectures.

### Retrospective analysis.

Results from the first round of analyses were compared with subsequent data from the Week 17 report. For N’Zoo, forecast cases could be directly compared with reported cases from the Week 17 report. For other subprefectures, the assessment was not as straightforward as the epidemics were not fully observed (i.e., underreported). Instead, we compare the forecasted number of cases for weeks 14–17 when the TSIR model is fit to the week 13 report, with the number of cases estimated to have occurred during those weeks when fitting the TSIR model to the week 17 report. We also fit an alternate TSIR model to the data, estimating the seasonality effect α based on data rather than fixing it.

As the case counts from the Week 13 and the Week 17 report are different in overlapping weeks, we consider how our results would have differed if the Week 13 report were the same as the corrected Week 13 report. We finally considered how the estimates for N’Zoo changed if we calculated the force of infection in a given week using numbers of infections from 1 week earlier, rather than 2.

## RESULTS

The Week 13 report gave observed measles cases in several subprefectures of Lola up to week 13 of 2015. N’Zoo subprefecture was the first to report cases of measles, beginning in the 4th week of the year. In the 9th week of the year, two cases were observed in Kokota subprefecture. The following week, cases were observed in N’Zoo and Kokota, along with a third subprefecture, Foumbadou; and by week 12, cases had been observed in eight subprefectures in total. Tounkarata subprefecture had four reported cases in week 15 and no other reported cases, and so is excluded from analysis entirely.

**Figure 2.**
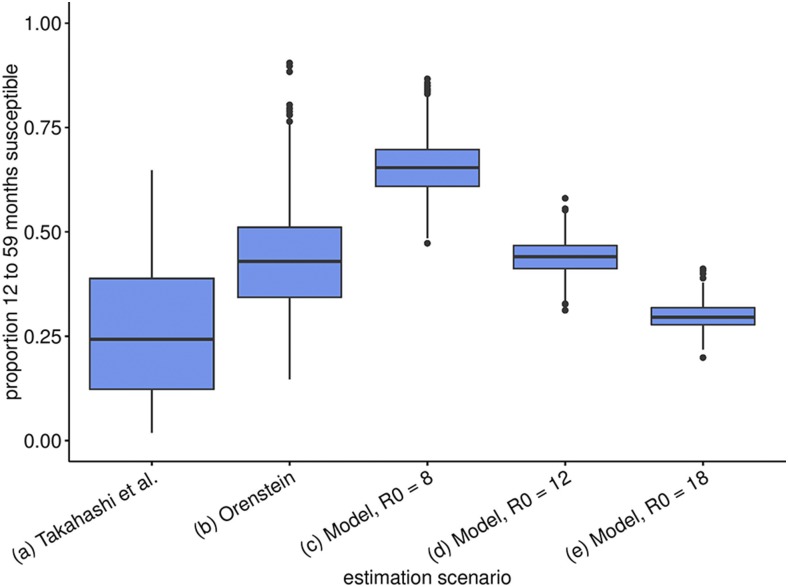
Expected susceptibility/percent unvaccinated. We use various fit scenarios and values of *R*_0_ for our time-series susceptible–infected–recovered method. Boxplot (a) shows the estimated proportion of individuals from 9 months to 5 years old who are susceptible, whereas boxplots (b–e) show the proportion of individuals from 1 year to 5 years old who are susceptible. Box plots give the interquartile range of estimates and whiskers the 95% credible interval of posterior estimates. This figure appears in color at www.ajtmh.org.

Figure 2 shows estimates of susceptibility in the 1- to 5-year-old age group in N’Zoo for our three methods. Estimating the proportion of susceptible individuals in the Nzérékoré region (which includes Lola prefecture), via the method of Takahashi et al., results in an estimated 24% (95% confidence interval [CI]: 2–65%) of children in the 9 months to 5-year-old age group being susceptible (Figure 2, boxplot [a]) (geo-located estimates for absolute numbers of susceptibles in this age group are shown in Figure 1). Results from Orenstein’s and TSIR methods consider the 1- to 5-year-old age group, as data were stratified into five nonoverlapping age groups: Less than 1 year, 1–5, 6–10, 11–15, and 16+ years olds. Using Orenstein’s method, we combined the proportion of cases occurring in vaccinated individuals with the assumed VE to estimate susceptibility and obtained a mean estimate of 43% (95% CI: 15–76%) (Figure 2, boxplot [b]).

Fitting N’Zoo incidence data for scenarios with *R*_0_ of 8, 12, and 18, using the TSIR method, we found that assuming a lower *R*_0_ led to a prediction of a larger epidemic than that for higher *R*_0_ values, as this required a higher level of susceptibility to explain observed cases (Figure 2, box plots [c]–[e]). The scenarios were thus designated as pessimistic, mid, and optimistic for the 8, 12, and 18 values of *R*_0_, respectively. Estimates from the optimistic scenario were mostly in line with estimates from other methods.

**Figure 3.**
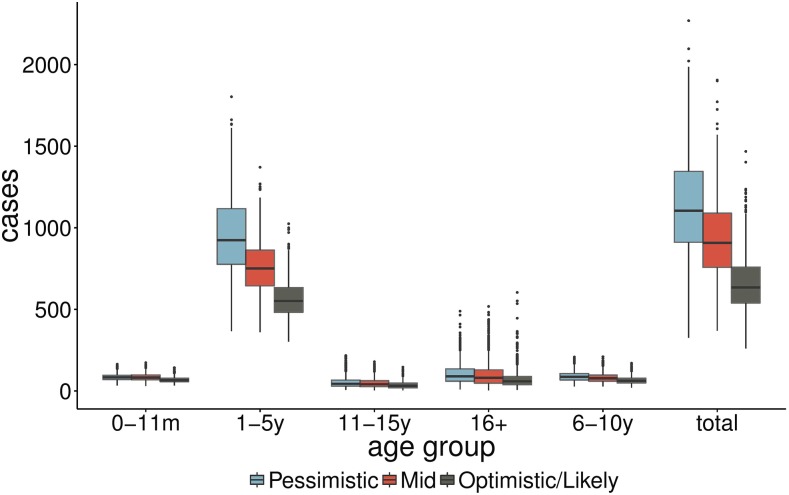
Predicted total cases up to Week 23 across age groups in N’Zoo subprefecture for three scenarios corresponding to *R*_0_ = 8, 12, and 18 (pessimistic, mid, and optimistic, respectively), using the Week 13 report to fit the model. Box and whisker plots represent interquartile range and 95% credible interval of posterior estimates. This figure appears in color at www.ajtmh.org.

**Figure 4.**
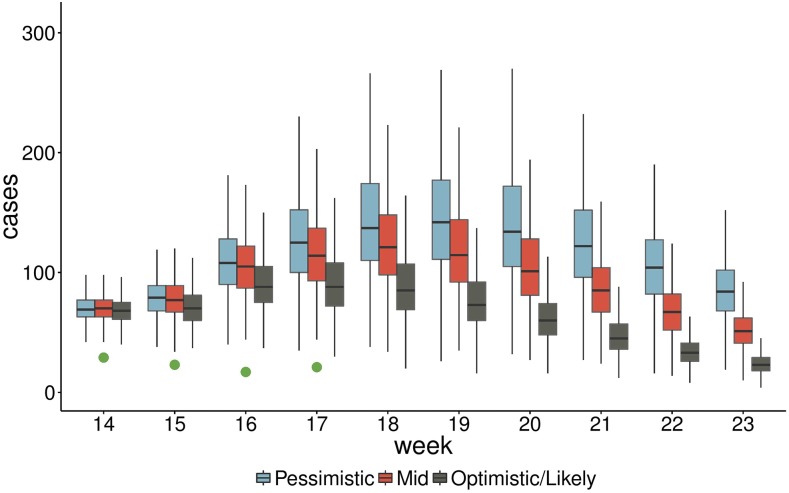
Predicted epidemic trajectory up to week 23 of 2015 for three scenarios corresponding to *R*_0_ = 8, 12, and 18 (pessimistic, mid, and optimistic, respectively). The green dots represent observed cases from weeks 14 to 17. Boxes represent the interquartile range and whiskers represent the 95% credible interval of posterior estimates. This figure appears in color at www.ajtmh.org.

Figure 3 shows estimates of cases by age group between weeks 14 and 23 of 2015 for N’Zoo only, whereas Figure 4 shows estimated cases by week over the same period, with the reported cases from weeks 14 to 17 also included (green dots). These were included in the first report that was sent to the ECDC and were shared with the World Health Organization.

These forecasts predicted a large outbreak of measles in N’Zoo, with an interquartile range of 539–759 total cases expected (mean 657) even in the optimistic scenario, with the vast majority of the cases in the 1- to 5-year olds.

Retrospectively, we compared estimates when the seasonality effect α is fixed across Lola with estimates when α is allowed to vary between subprefectures. These estimates are shown in Table 1, where we compare parameter estimates from this model for each subprefecture. The first two columns give the parameter being estimated and the subprefecture in question, respectively. The final three columns of the table give estimates from the model when we use, in turn, the Week 13 report, the corrected Week 13 report, and the Week 17 report to fit the model. For all subprefectures except N’Zoo, the posterior means for α were between 0.41 and 0.44 (95% CI: 0.32–0.54), implying a stronger seasonal signal than was initially assumed. In N’Zoo, the posterior mean was 0.31 (95% CI: 0.22–0.43). The overall size of the outbreak is forecast to be smaller if seasonality is stronger, as from week 14 onward there is a greater decrease in transmission due to this strong seasonal effect (Supplemental Figure 1).

**Table 1:** Estimated parameters from time-series susceptible–infected–recovered–fit model where the seasonal effect α is a parameter fit by the model

Parameter	Location	Week 13 report—mean (95% confidence interval [CI])	Corrected Week 13 report—mean (95% CI)	Week 17 report—mean (95% CI)
Basic reproduction number *R*_0_	N’Zoo	20.19 (15.93–24.96)	18.73 (14.55–23.57)	16.41 (12.56–21.07)
	Foumbadou	11.09 (8.41–14.27)	14.61 (11.14–18.47)	15.69 (12.58–19.15)
	Gueasso	13.38 (9.82–17.38)	17.50 (13.79–21.51)	11.64 (9.08–14.79)
	Gama Berema	13.59 (9.72–17.93)	18.40 (14.48–22.44)	12.02 (9.52–14.76)
	Kokota	15.80 (10.97–20.46)	15.11 (12.61–19.63)	16.28 (12.73–19.66)
	Laine	18.48 (14.32–22.67)	19.56 (14.76–24.19)	17.16 (14.42–19.90)
Less than 1 year’s susceptibility	N’Zoo	42% (25–63%)	31% (17–50%)	21% (13–33%)
	Foumbadou	51% (37–68%)	37% (24–50%)	26% (19–35%)
	Gueasso	37% (19–55%)	24% (10–39%)	15% (7–21%)
	Gama Berema	36% (20–54%)	29% (16–44%)	18% (11–27%)
	Kokota	45% (30–62%)	18% (0–38%)	10% (1–15%)
	Laine	41% (24–60%)	33% (18–48%)	19% (11–30%)
1–5’s susceptibility	N’Zoo	30% (25–35%)	29% (24–35%)	27% (22%–33%)
	Foumbadou	26% (21–31%)	27% (22–32%)	23% (19–27%)
	Gueasso	26% (21–31%)	28% (23–32%)	25% (20–28%)
	Gama Berema	26% (21–31%)	29% (24–35%)	25% (21–29%)
	Kokota	27% (22–32%)	27% (22–31%)	29% (24–35%)
	Laine	29% (24–34%)	29% (23–34%)	27% (13–26%)
6–10’s susceptibility	N’Zoo	18% (10–29%)	18% (9–30%)	19% (12–28%)
	Foumbadou	9% (1–21%)	13% (1–24%)	15% (9–22%)
	Gueasso	17% (9–27%)	19% (11–28%)	20% (15–27%)
	Gama Berema	12% (2–23%)	12% (2–23%)	14% (8–20%)
	Kokota	10% (0–22%)	19% (12–26%)	20% (15–27%)
	Laine	15% (4–25%)	17% (6–27%)	19% (13–26%)
11–15’s susceptibility	N’Zoo	8% (2–19%)	4% (0–10%)	6% (2–12%)
	Foumbadou	8% (2–13%)	5% (2–10%)	8% (5–12%)
	Gueasso	5% (0–13%)	3% (0–8%)	0% (0–1%)
	Gama Berema	5% (0–13%)	3% (0–8%)	1% (0–5%)
	Kokota	2% (0–11%)	1% (0–7%)	0% (0–2%)
	Laine	6% (0–14%)	4% (0–9%)	2% (0–8%)
16+ susceptibility	N’Zoo	3% (1–6%)	3% (1–7%)	2% (1–4%)
	Foumbadou	1% (1–3%)	2% (0–5%)	1% (0–2%)
	Gueasso	2% (0–4%)	2% (0–4%)	0% (0–1%)
	Gama Berema	3% (1–5%)	3% (1–5%)	1% (0–3%)
	Kokota	1% (0–3%)	0% (0–3%)	2% (0–3%)
	Laine	2% (0–5%)	3% (0–5%)	1% (0–2%)
Seasonal effect α	N’Zoo	0.31 (0.21–0.46)	0.33 (0.21–0.49)	0.45 (0.39–0.59)
	Foumbadou	0.44 (0.32–0.56)	0.44 (0.28–0.57)	0.48 (0.34–0.59)
	Gueasso	0.44 (0.31–0.56)	0.44 (0.31–0.56)	0.46 (0.33–0.57)
	Gama Berema	0.45 (0.32–0.56)	0.44 (0.31–0.56)	0.45 (0.32–0.57)
	Kokota	0.44 (0.32–0.56)	0.43 (0.31–0.53)	0.51 (0.34–0.58)
	Laine	0.44 (0.32–0.56)	0.45 (0.31–0.57)	0.43 (0.31–0.55)
Reporting rate δ	Foumbadou	8% (5–13%)	14% (8–27%)	20% (16–26%)
	Gueasso	14% (8–25%)	32% (23–43%)	17% (14–22%)
	Gama Berema	16% (10–26%)	36% (22–69%)	17% (13–22%)
	Kokota	40% (5–100%)	6% (5–9%)	6% (5–9%)
	Laine	7% (5–14%)	7% (5–12%)	6% (5–8%)

The final three columns describe which data were used to fit the model.

**Figure 5.**
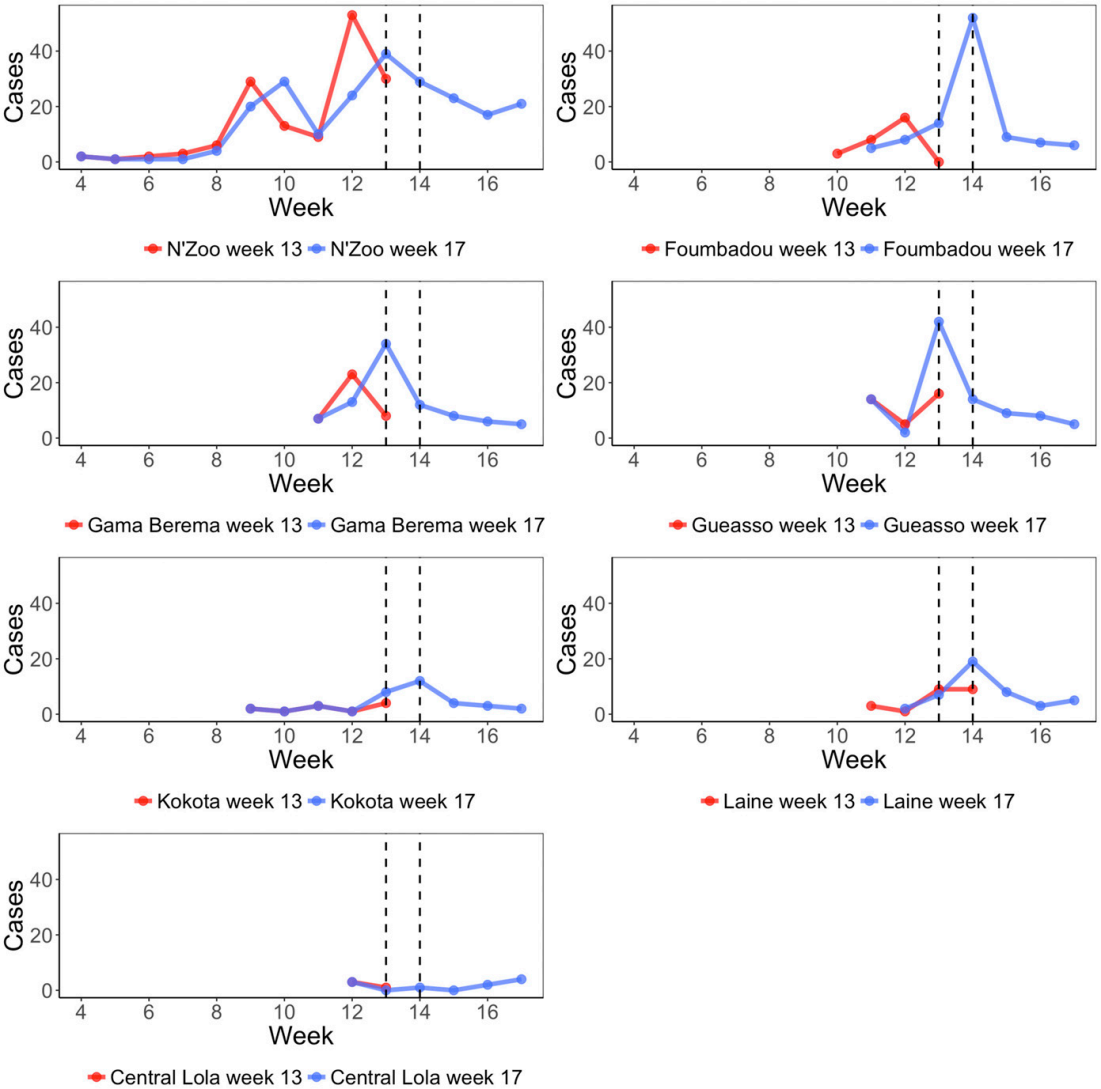
Reported cases by week in the seven subprefectures of Lola prefecture, with the data from the week 13 report shown in red and data from the week 17 report shown in blue. We see a disagreement in the cases reported in the overlapping weeks from both reports, in some cases the beginning of the epidemic being in different weeks. In addition to cases in these subprefectures, there were also four cases reported in Tounkarata subprefecture in week 15, which are not displayed here. The two vertical lines indicate when data were received and when forecasts were provided to collaborators in Guinea, respectively. This figure appears in color at www.ajtmh.org.

To gauge the accuracy of forecasts, we compare with the Week 17 report. As in N’Zoo, we assume that the outbreak was almost fully reported. We can compare our forecasts from the Week 13 report directly with the number of cases reported in the Week 17 report. We see that estimates of the number of cases between weeks 14 and 17 were far higher than what was observed (Figure 5).

In the other subprefectures, as we do not assume complete observation, we instead compare results from the Week 13 report with those when using the Week 17 report. We find a good agreement between the results produced from analyzing both data sets (Figure 5). Central Lola was excluded from this analysis as the number of cases reported was insufficient for the models to fit.

To understand to what degree errors in our predictions were due to differences in the reported number of cases up to week 13 between the Week 13 and Week 17 reports, we examined the forecasts to week 17 that we would have made had our data at week 13 been consistent with the corrected Week 13 report (comparisons of predictions by week are shown in Figure 5). In Table 2, we compare estimates for the attack rate in each subprefecture up to week 17 when using the Week 13 report, the corrected Week 13 report, and the Week 17 report. For N’Zoo, Foumbadou, and Laine, we see that if the Week 13 report were in agreement with the corrected Week 13 report, then our initial predictions would have been closer to the later predictions. However, in the other subprefectures, the results would have been further from later predictions.

**Table 2:** Estimated attack rates per 1,000, across the entire population, up to Week 17 of 2015 by subprefecture and data on which predictions are based on

Subprefecture	Week 13 report	Corrected Week 13 report	Week 17 report
N’Zoo	32.6 (22.0–48.4)	17.7 (13.0–25.5)	14.2
Foumbadou	37.2 (25.2–50.5)	32.7 (20.6–47.4)	29.0 (20.6–34.0)
Gueasso	30.6 (20.1–43.7)	36.5 (25.2–52.3)	26.7 (27.3–28.2)
Gama Berema	31.9 (21.7–44.7)	37.6 (23.7–53.5)	24.8 (19.0–28.6)
Laine	47.7 (31.3–64.0)	32.8 (16.1–54.4)	20.8 (13.0–29.2)
Kokota	22.8 (3.1–42.5)	34.8 (27.5–49.4)	39.0 (36.7–43.0)

We assume that N’Zoo is fully observed up to week 17; therefore, the Week 17 report result has no credible intervals.

**Figure 6.**
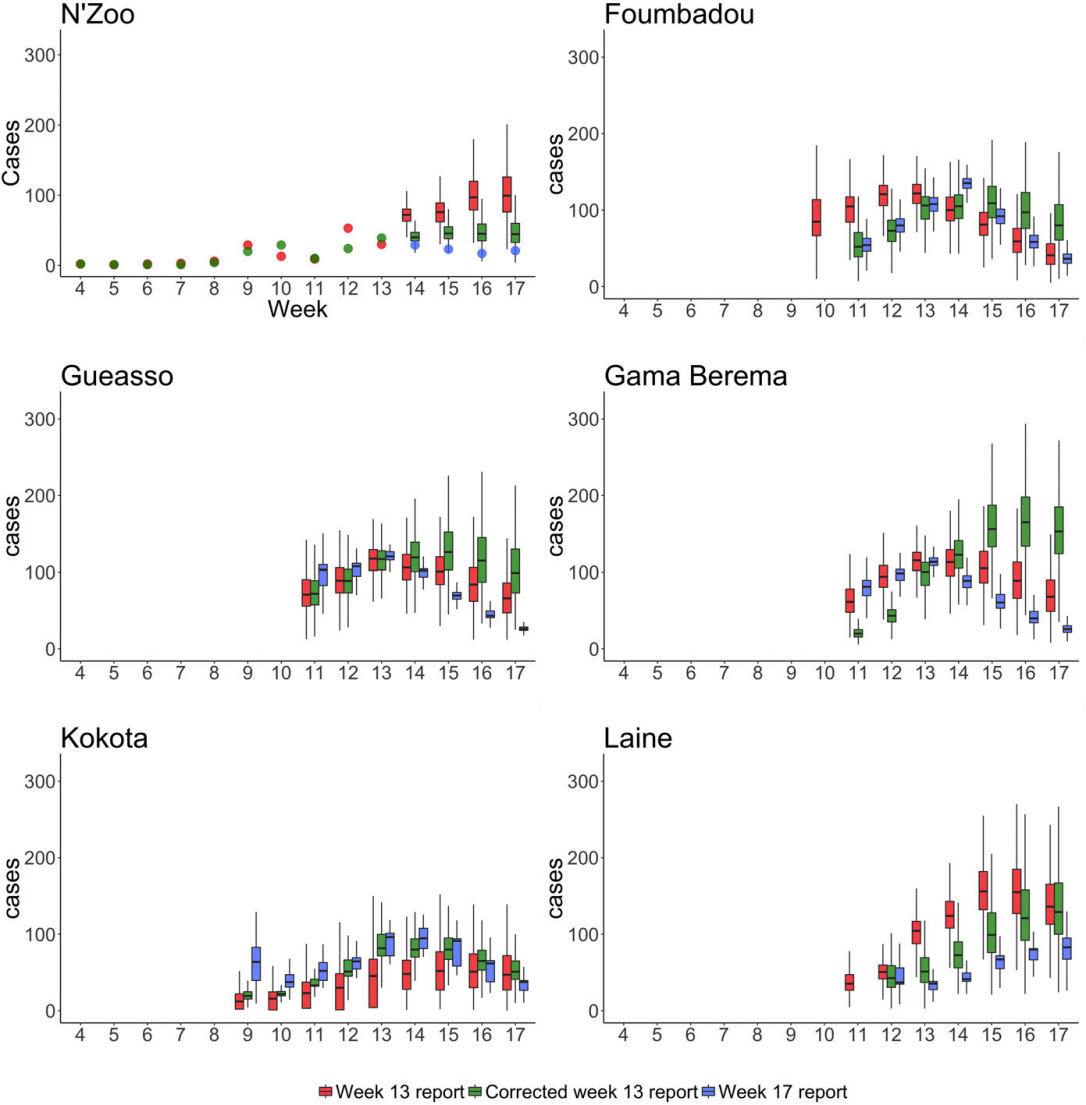
Predictions of total cases in six subprefectures when *R*_0_ and a are allowed to vary. We plot the predictions made when using the Week 13 report in red, predictions from the corrected Week 13 report in green, and those from the Week 17 report in blue. For N’Zoo, we assume that the observed cases are equal to all cases; therefore, we plot the data from the Week 13 and Week 17 reports as dots. Because of the lack of data (see Figure 7), the estimates for Central Lola subprefecture were poor (because of non-converging Markov chain Monte Carlo chains) and so are not displayed here. Boxes represent the interquartile range and whiskers represent the 95% credible interval of posterior estimates. This figure appears in color at www.ajtmh.org.

We also considered sensitivity to model specification for N’Zoo. Initially, a 2-week time step was used in the TSIR model here, whereas in other subprefectures, because of the lack of data, a 1-week case dependence was used. Figure 6 shows case predictions up to week 17 when we use cases from 1 week and 2 weeks earlier to calculate the force of infection to fit the data from the Week 13 report in the TSIR framework. The mean estimate for the cumulative cases through week 17 of the year with a 2-week dependence was 508, whereas when using a 1-week dependence this was down to 302, more in line with the number of reported cases, 221.

Although absolute numbers differed, the relative number of reported cases by age group for each subprefecture was a good match for our predictions, indicating that the estimates of relative susceptibility by age was in line with reality (Supplemental Figures 2 and 3).

## DISCUSSION

Here we describe an analysis of a measles outbreak in Lola prefecture of Guinea beginning in the 4th week of 2015, including estimates of population susceptibility and projection of future cases in the Nzérékoré region and Lola prefecture using several methods of different complexity. Projections made from the Week 13 report were supplied to the WHO on April 12, 2015. A measles vaccination campaign deployed to compensate for the interrupted Guinea-wide campaign originally planned for 2014 began in Lola prefecture on April 18 (week 16). This campaign was conducted over 7 days (the entirety of week 17), and it is estimated that ultimately 92% of children from 6 months to 10 years old were vaccinated.^4^ Despite this vaccination effort, the area has remained vulnerable to measles, as demonstrated by the subsequent measles outbreak in Guinea in 2017. More than 2,100 children were infected with measles from January 2017 to March 12, 2017, with 675 of these cases reported in Nzérékoré prefecture^20^ (located within the Nzérékoré region and bordering Lola prefecture), a greater number of cases than were reported in Lola in 2015. A vaccination campaign was initiated on March 12, 2017, in Nzérékoré prefecture, aimed at protecting 140,000 children between the ages of 6 months and 10 years.

In general, our models projected a greater number of cases than were reported. Our analyses considered two different data reports: one provided in week 13 of the outbreak and one in week 17, termed the Week 13 and Week 17 reports, respectively. For N’Zoo, where cases were assumed to be fully reported, our predictions for weeks 14–17 made from the Week 13 report were higher than those observed in the subsequent report. Outside of N’Zoo, as it was assumed that cases were underreported, we cannot directly compare our forecasts with reported cases to assess accuracy. However, the predictions made for weeks 14–17 with the Week 13 report were generally consistent with model fits of this period using the Week 17 report. In N’Zoo, analyses using the data from the corrected Week 13 report improved forecasts, suggesting that data issues hampered forecasts here; however, this worsened forecasts for other subprefectures. Outside of N’Zoo, the estimated peak week of the epidemic when using the Week 13 report was generally closer to the data and the estimate when using the Week 17 report, than it was when using the corrected Week 13 report (Table 3).

**Table 3:** Peak week of the epidemic by subprefecture according to data from the Week 17 report, and model fits using the Week 13, corrected Week 13, and Week 17 reports

Subprefecture	Data	Week 13 report	Corrected Week 13 report	Week 17 report
N’Zoo	13	> 17	15	NA
Foumbadou	14	13	15	13
Gueasso	13	13	15	13
Gama Berema	13	13	16	13
Laine	14	15	13	13
Kokota	14	16	> 17	> 17

NA = not applicable. N’Zoo has NA for the estimate from the Week 17 report, as the model was never run for the full Week 17 report, as observation was assumed to be complete. Entries that are “> 17” imply that the number of cases were estimated to still be increasing at week 17.

**Figure 7.**
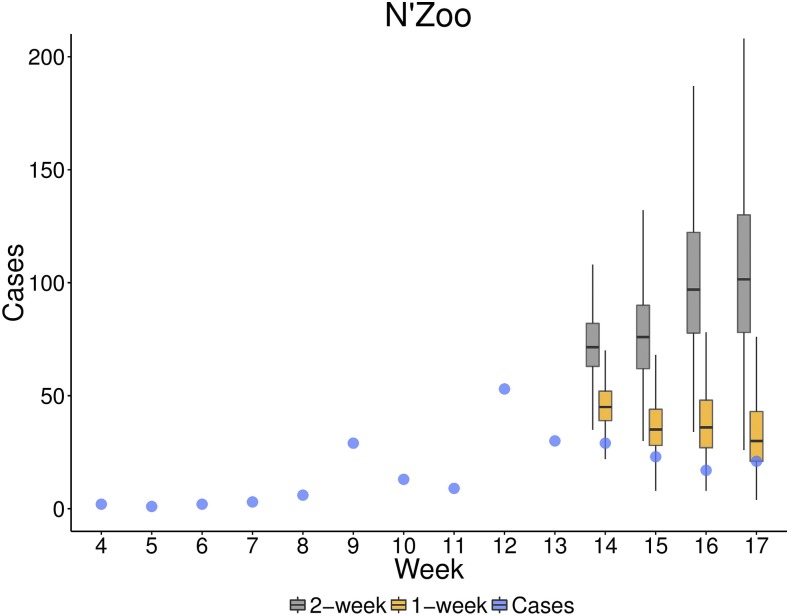
Predicted cases in weeks 14–17 from fitting time-series susceptible-infected-recovered (TSIR) models for N’Zoo when *R*_0_ and a are allowed to vary using 1- and 2-week time steps to the Week 13 report. The reported cases in N’Zoo are given by blue dots. Box plots indicate the interquartile range of posterior estimates. Predictions when we use a 2-week time step in the TSIR model are plotted in gray and when using a 1-week time step in yellow. Boxes represent the interquartile range and whiskers represent the 95% credible interval of posterior estimates. This figure appears in color at www.ajtmh.org.

It is possible that surveillance changed over time and that this was responsible for the peak in cases around week 13, followed by a precipitous drop, which was seen in all subprefectures except N’Zoo (Figure 7). This pattern is hard to reproduce with any epidemic model, so it seems likely that changes in surveillance or behavioral changes in advance of the start of vaccination might have modified the dynamics in ways unaccounted for by the models.

As well as the impact of data variation, using different model variants produced different forecasts. Fitting the seasonal fluctuations in transmission resulted in a decrease in the predicted cases. Furthermore, comparisons over models fitted to the N’Zoo data implied that a 1-week rather than a 2-week case dependence gave better predictions over the subsequent weeks of the epidemic. This may be due to nonconstant serial intervals among individuals,^17,18^ which our initial model assumed to be 2 weeks across all individuals.

Initial estimates for the attack rate up to week 17 were broadly in agreement across subprefectures, with Kokota and Laine being on the low and high end of the estimates, respectively. In particular, N’Zoo’s estimated attack rate up to week 17 was approximately equal to that of Foumbadou, Gueasso, and Gama Berema. Using the corrected Week 13 report, the estimated attack rate up to week 17 was lower in N’Zoo (attack rate of 14.2 per 1,000 down from 32.7 per 1,000 using the Week 13 report) than in all other subprefectures (estimated attack rates ranging from 20.6 to 38.9 per 1,000). This may be because of the fact that the surveillance process and the epidemic process are not independent of each other; therefore, the highly effective surveillance of the local health workers in N’Zoo may have led to a more effective response, significantly curtailing the epidemic. In addition, this may indicate a bias in the initial reporting, which was mitigated when data were updated. It may also follow that if other subprefectures had good data, then a decrease in the attack rate may also have been seen for these places.

This analysis highlights the uses and limitations of “just-in-time” modeling studies in outbreak response. This outbreak was in some ways a special case, as the Ebola outbreak had disrupted routine vaccination and interrupted a scheduled vaccination campaign. In addition, routine disease surveillance, along with many other public health activities, had been negatively affected, as virtually all focus was directed toward responding to the Ebola crisis. For these reasons, a measles vaccination campaign was long overdue, and the outbreak served to highlight its necessity. Generally during outbreaks, modeling can be useful for motivating action and preventing many additional cases^21,22^—indeed, in the situation described, the modeling efforts may in part have motivated a campaign de novo. Modeling can also be used to help plan vaccination campaigns by characterizing the susceptibility of the population in different areas, thereby helping to focus initial efforts. As demonstrated by the results from refitting models with the corrected Week 13 data, updating analyses when corrected data becomes available can potentially lead to improvements in estimates and hence conclusions and recommendations, when compared with using a static system which is not updated over time.

Our forecasts may have overestimated the number of cases by 1.5–3 times (Table 1). Discrepancies between forecasts and observed cases were likely a result of a combination of factors, including inaccurate and inconsistent reporting of case numbers across reports, selection of models that were necessarily underpowered given the length of the time series available, and the effects of interventions and changing surveillance patterns on the observed course of the epidemic. Overcoming these challenges is not something that can take place over the course of a single outbreak, but requires long-term improvements in the storage, dissemination, and handling of infectious disease data so that when a crisis does occur, information is available to formulate an appropriate response. Not all these limitations can be overcome, nor do they necessarily indicate a shortcoming of model forecasts; in particular, model forecasts may lead to subsequent interventions that change the course of the epidemic, rendering the forecasts themselves invalid.^23–25^

Although forecasting can be a useful stand-alone tool, these analyses can shed light more broadly on the underlying processes that generated the observed epidemic data. Here, and in other settings, models provided a picture of what was driving the outbreak: high measles susceptibility in the region. Although the point estimates of measles susceptibility from our three methods diverged, all three provided a consistent picture of at least 24% of children in Lola prefecture being unvaccinated for measles, consistent with previous estimates in the Nzérékoré region.^2^ Models further provide plausible planning scenarios based on current information and can be important tools for health officers on the ground.^25^ In this case, although projections of case numbers ultimately were found to be overestimates, they provided a correct order-of-magnitude picture of what could be expected even in the worst plausible scenarios. Given the uncertainties in outbreaks, particularly where there is few prior data, this may set a practical limit on expectations from modeling in this context, and model forecasts should be presented and interpreted with caution.^26^

For the analysis and forecast team, the main difficulties of this collaboration were the quick turnaround in estimates and predictions, which were required and thus generated in little over 1 week. This involved construction of models from the ground up, followed by fitting parameters and generating forecasts, leaving little time to consider different model formulations that may have resulted in improved forecasts. In future, having multiple models developed and available for forecasting before an outbreak occurs would aid the production of time-sensitive forecasts.

Challenges faced by the team in the field were predominantly related to the logistics of obtaining regular data updates and to their uncertainties surrounding the quality of the reported data. Given the outbreak situation across multiple subprefectures that each required at least half a day to visit, it was not feasible or possible for the team to validate all reported data as they came in; some data were only able to be validated several days after the initial reporting. Another challenge related to the ad hoc establishment of this project: the field team was required to try to learn on the fly the specific modeling methodologies being deployed. In an ideal situation, a relationship between a field and a modeling team would be preestablished to ensure that all parties were thoroughly familiar with the key model inputs, uncertainties, and outputs. Nonetheless, the initial modeling results were very useful for the field team to understand the potential trajectories of the outbreak and to advocate for action and collaboration with local and international partners.

From a longer term perspective, enhanced collaboration between modelers and field epidemiologists would be best achieved in non-outbreak situations as part of emergency preparedness planning. Necessary understanding about a range of issues could then be discussed and agreed upon. These would include data usage and privacy, the types of data likely to be available during an outbreak situation, and the nuances of specific modeling approaches in terms of key data requirements, limitations, and uncertainties.

In every emerging disease crisis, decisions are made in highly uncertain situations, and making full use of the data is critical to making the best possible decisions as to how to respond. Modeling approaches that provide both situational awareness and practical projections for planning purposes can help, but are best positioned as part of an iterative collaboration between analysts and those setting policy in the field, where forecasts can be updated as new data are collected. The Lola prefecture experience illustrates some of the challenges and benefits of this approach and highlights the need for established tools and partnerships to address future outbreaks.

## Supplementary Material

Supplemental Information and Figure.
