# Supplementary material for: Challenges and Opportunities in Disease Forecasting in Outbreak Settings: A Case Study of Measles in Lola Prefecture, Guinea
Source: Am J Trop Med Hyg. 2018 Mar 12;98(5):1489–97. doi: 10.4269/ajtmh.17-0218 (PMC5953353; doi:10.4269/ajtmh.17-0218)
Supplement: Supplementary file 1 [file tpmd170218.SD1.pdf]

SUPPLEMENTAL INFORMATION

Methods for susceptibility estimation. Three methods were used to estimate the susceptibility of the population.

Orenstein's method.[5] The proportion of cases occurring in vaccinated individuals (PCV) and VE (assumed to be between 86% and 97%) are used to estimate the PPV (and hence immune) solely based on case data.

The PPV is estimated to be PPV = PCV/(1 − VE [1 − PCV]). A uniform distribution between 0.86 and 0.97 was assumed for VE and the data were bootstrapped to give an estimate of the distribution of PPV.

Method of Takahashi et al. Geo-located data from Demographic and Health Surveys[9] were used to estimate the rate of vaccination of children before the impact of Ebola. To approximate the impact made by Ebola on vaccination, a 25% reduction in this rate was assumed for the duration of the Ebola outbreak. This method results in an estimated susceptibility for children up to the age of 5 years in the population at the beginning of the measles outbreak. This method was developed by Takahashi et al.[1]

Discrete time SIR model. Weekly incidence data were split into five nonoverlapping age groups: Less than 1, 1–5, 6–10, 11–15, and 16+ years olds. The model was seasonally forced and aimed to estimate the susceptibility to measles at the beginning of the outbreak in each of these age groups. Births were not included in the model because of the short timescale over which the outbreak took place. The basic goal was to estimate the number of susceptible individuals in each age group a at time 0, which is denoted by $S(a,0)$. We can then calculate susceptibility at subsequent times by subtracting observed or suspected cases from this initial value, so the number of susceptibles in week w is given as follows:

$$S(a,w) = S(a,0) - (I(a,1) + \ldots + I(a,w-1)).$$

The seasonality was modeled by the equation $\beta(t)$ = mean(β) $(1 + \alpha \cos[2\pi t])$, where β is the transmission parameter, which is dependent on R_0, and α is the amplitude of the seasonal effect on transmission. This form of seasonality and the timing of peaks and troughs of the pattern were chosen to match historical measles cases in Guinea. For initial analysis, the value of α was kept constant at 0.3. This was later treated as a parameter of the model and allowed to vary between 0.2 and 0.6.

Uniform mixing between age groups and assortative mixing were considered. When comparing the results gained using this method with those of other methods, a mixing matrix based on the total number of contacts found in Read et al.[27] gave the strongest agreement and so was used throughout.

The force of infection was calculated using the next-generation method.[28]

Model fitting for N'Zoo. Initially we assumed a fixed value of R_0. We considered scenarios where R_0 was 8, 12, and 18 (estimates of R_0 typically range from 12 to 18,[12] although values as low as 6 have been estimated[13]).

To approximate the serial interval, it was assumed that cases in week $w+2$ were dependent on cases in week w. This paradigm was chosen over the more common biweekly approach, where cumulative cases in a 2-week period are dependent on the cumulative cases over the previous 2 weeks, to maximize the utility of available data given a serial interval of approximately 2 weeks. Therefore, the force of infection on each age group a in week $w+2$ is based on cases from week w and denoted by $\phi(a,w)$.

We assume that cases of measles in week $w + 2$ were Poisson distributed as follows:

$$I(a, w+2) \sim \text{Poisson}(\phi(a,w)S(a,w)),$$

where $S(a,w)$ is the number of susceptible individuals for age group a in week w, $I(a,w + 2)$ is the number of infected individuals for age group a in week $w+2$, and $\phi(a,w)$ is the force of infection for age group a based on cases in week w. A Poisson approximation to the binomial distribution was used for computational efficiency.

Initial analysis assumed that R_0 was known (either 8, 12, or 18, given which scenario was being considered), along with the amplitude of the seasonality, α, which therefore defined the force of infection given the number of infected individuals each week. In this case, the only unknown parameter was the initial susceptibility, $S(a,0)$, which was fit using Markov chain Monte Carlo methods in RStan. From this, we can calculate the remaining number of susceptibles each subsequent week. Subsequent analysis treated R_0 and α as parameters to be fit along with the initial susceptibility.

Forecasting N'Zoo. To forecast upcoming cases, values of the susceptibility for each age group were sampled from the posterior, and the cases up to week 13 were subtracted from the number of susceptibles this implied. The force of infection was then calculated using the average of the previous 2 weeks of the epidemic to give the initial number of infected individuals. The number of cases in the following week was then chosen from a binomial distribution with $n = S(a,w)$ and $p = \phi(a,w)$. Once this was sampled, we then averaged this with the number of infected individuals in the previous time step to get the new number of infected individuals and continue on in this manner, until we have projected cases as far forward as desired. This was repeated 1,000 times.

Model fitting for other subprefectures. In other subprefectures, cases of measles were likely significantly underreported. Few cases were officially reported, whereas pharmacies from the area reported seeing far greater numbers of people with measles-like symptoms. To account for this, the model was adjusted to include a parameter δ modeling the observation process. This was modeled so that the value of δ gives the proportion of cases in the outbreak that were observed.

Many of the subprefectures had only 2 or 3 weeks of data on cases. Therefore, because of the lack of data, a 1-week case dependence was used rather than 2 weeks.

Again, the force of infection was calculated using the next-generation method.[28] To distinguish it from $\phi(a,t)$ from the N'Zoo model, we denote it by $\psi(a,t)$, as it has a different interpretation when using the 1-week instead of 2-week case dependence. Rather than using a scenario-based analysis, by selecting values of R_0, this is treated as a parameter to fit in this model. Again, initially the amplitude of the seasonality, α, was assumed to be constant at 0.3 and later was allowed to vary between 0.2 and 0.6.

Cases of measles in week $w + 1$ were assumed to be Poisson distributed as follows:

$$I(a, w+1) \sim \text{Poisson}(\psi(a,w)\,S(a,w)).$$

Data, giving observed cases in each age group *a* by week *w*, denoted by $C(a,w)$, are assumed to be binomially distributed, with $n = I(a,w)$, the number of infections in each age group *a* by week *w*, and $p = \delta$, the parameter modeling the observation process. For ease of computation, we estimate the binomial distribution by a normal distribution as follows:

$(a,w) \sim \text{Normal}\ (\mu = \delta\ I(a,w),\ \sigma^2 = \delta(1 - \delta)\ I(a,w))$. Posterior distributions from the N'Zoo model for susceptibility and R_0 were used as earlier here.

We assumed that all subprefectures in the area have fairly similar characteristics. Therefore, we would expect to see similar levels of susceptibility in the age groups in each subprefecture and would expect to see incidence levels that were comparable with those of N'Zoo. Consequently, δ was assumed to be at least 5% and infections in any specific age group (given by $I(a,w)$) were constrained to be less than 100 per week, as the maximum number of cases in any age group in 1 week for N'Zoo was 41.

Forecasts for other areas were made similarly as for N'Zoo, except that estimated infections from the model were subtracted from initial susceptibles rather than reported cases.

SUPPLEMENTAL REFERENCES

27. Read JM, Lessler J, Riley S, Wang S, Tan LJ, Kwok KO, Guan Y, Jiang CQ, Cummings DA, 2014. Social mixing patterns in rural and urban areas of southern China. *Proc Biol Sci 281:* 20140268.
28. Klepac P et al., 2009. Stage-structured transmission of phocine distemper virus in the Dutch 2002 outbreak. *Proc Biol Sci 276:* 2469–2476.

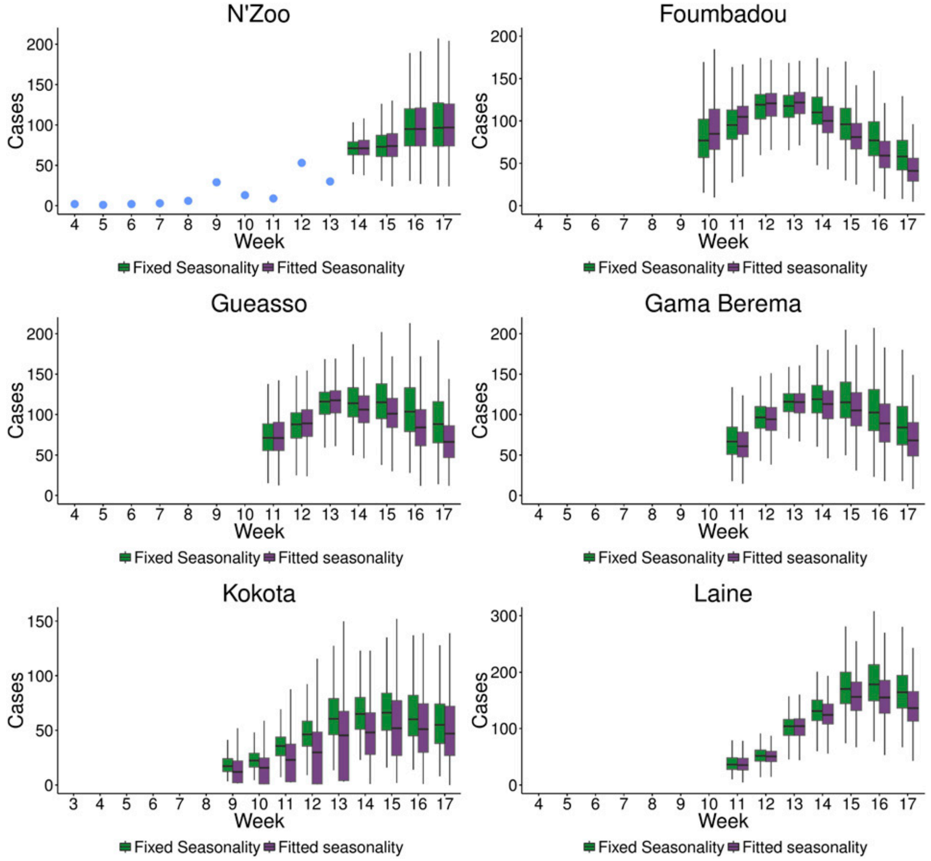

Supplemental Figure 1. A comparison of fits to the data, with fixed seasonality effect and where this is estimated. Estimates when seasonality is xed are shown in green and estimates when this is fit in the time-series susceptible–infected–recovered model are in purple. We predicted slightly fewer cases when the seasonality was allowed to vary. For N'Zoo, the blue dots represent reported cases, which are assumed to capture all cases; therefore, we plot the data for weeks 4–13. For other subprefectures, the reported data are assumed to be underestimates, so we plot posterior estimates for all weeks. Boxes represent the interquartile range and whiskers represent the 95% credible interval of posterior estimates.

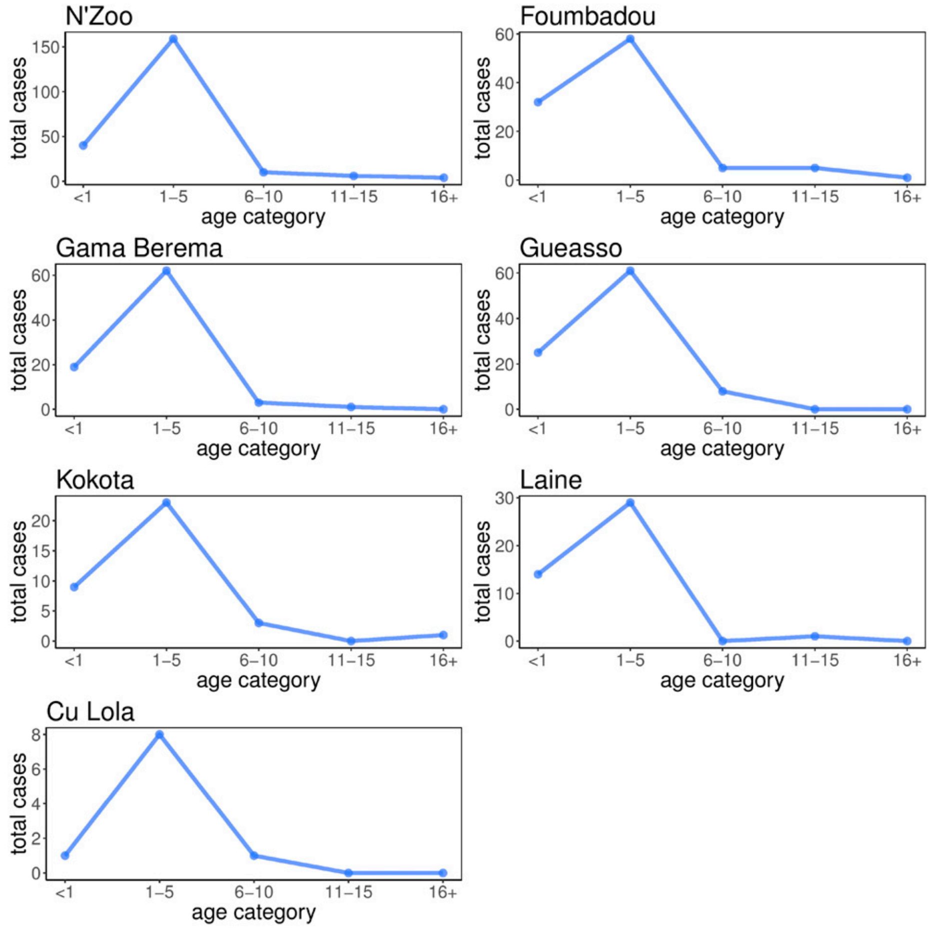

Supplemental Figure 2. Total reported cases in seven subprefectures in Lola prefecture, by age category from the beginning of the outbreak until week 17.

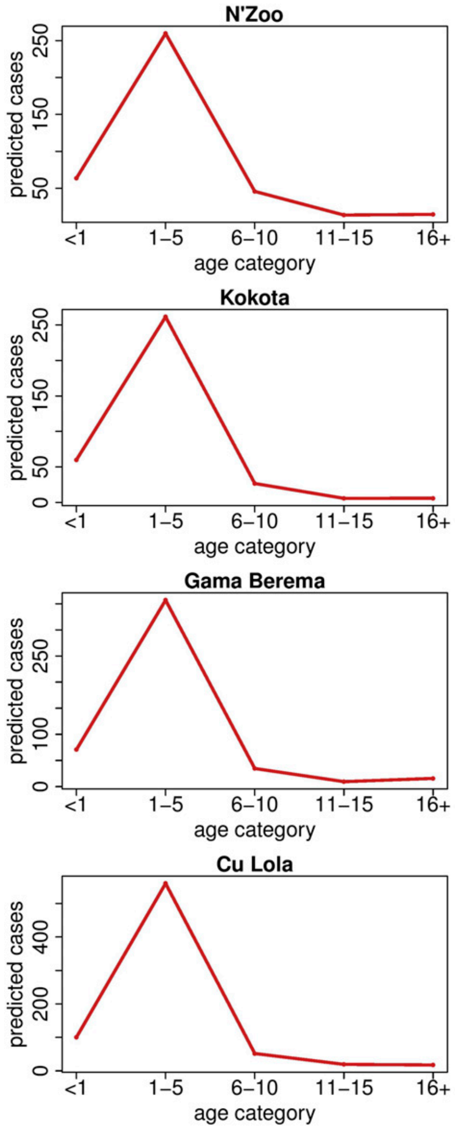

Supplemental Figure 3. Total predicted cases in seven subprefectures in Lola prefecture, by age category from the beginning of the outbreak until week 17.
